# C-Terminal Domain Swapping of SSB Changes the Size of the ssDNA Binding Site

**DOI:** 10.1155/2014/573936

**Published:** 2014-08-04

**Authors:** Yen-Hua Huang, Cheng-Yang Huang

**Affiliations:** ^1^School of Biomedical Sciences, Chung Shan Medical University, No.110, Sec.1, Chien-Kuo N. Rd., Taichung City, Taiwan; ^2^Department of Medical Research, Chung Shan Medical University Hospital, No.110, Sec.1, Chien-Kuo N. Rd., Taichung City, Taiwan

## Abstract

Single-stranded DNA-binding protein (SSB) plays an important role in DNA metabolism, including DNA replication, repair, and recombination, and is therefore essential for cell survival. Bacterial SSB consists of an N-terminal ssDNA-binding/oligomerization domain and a flexible C-terminal protein-protein interaction domain. We characterized the ssDNA-binding properties of *Klebsiella pneumoniae* SSB (KpSSB), *Salmonella enterica* Serovar Typhimurium LT2 SSB (StSSB), *Pseudomonas aeruginosa* PAO1 SSB (PaSSB), and two chimeric KpSSB proteins, namely, KpSSBnStSSBc and KpSSBnPaSSBc. The C-terminal domain of StSSB or PaSSB was exchanged with that of KpSSB through protein chimeragenesis. By using the electrophoretic mobility shift assay, we characterized the stoichiometry of KpSSB, StSSB, PaSSB, KpSSBnStSSBc, and KpSSBnPaSSBc, complexed with a series of ssDNA homopolymers. The binding site sizes were determined to be 26 ± 2, 21 ± 2, 29 ± 2, 21 ± 2, and 29 ± 2 nucleotides (nt), respectively. Comparison of the binding site sizes of KpSSB, KpSSBnStSSBc, and KpSSBnPaSSBc showed that the C-terminal domain swapping of SSB changes the size of the binding site. Our observations suggest that not only the conserved N-terminal domain but also the C-terminal domain of SSB is an important determinant for ssDNA binding.

## 1. Introduction

Single-stranded DNA-binding protein (SSB) specifically binds to single-stranded DNA (ssDNA) and is known to have important functions in the DNA metabolic processes, such as DNA replication, repair, and recombination of both prokaryotes and eukaryotes [1–4]. During these reactions, SSB binds to and protects susceptible ssDNA from nucleolytic digestion and chemical attacks and also prevents secondary structure formation [5]. Many but not all bacterial and human mitochondrial SSBs are active as homotetramers [5–7], in which four oligonucleotide/oligosaccharide-binding folds (OB folds) form a DNA-binding domain [8–12]. However, SSB from the bacterial phylum Deinococcus-Thermus functions as a homodimer, in which each monomer contains two OB folds linked by a conserved spacer sequence [13–20]. SSB from* Sulfolobus solfataricus* is a monomer that includes one OB fold, which differentiates SSB from the bacterial form, and is likely to be a more ancestral “simple” SSB [21–25]. The DdrB protein from* Deinococcus radiodurans* is an alternative SSB and functions as a pentamer [26]. Recent studies found that a distinct SSB from hyperthermophilic Crenarchaea, termed ThermoDBP, has ssDNA-binding domains that are markedly different from the classical OB folds of bacterial SSB [27, 28].

Bacterial SSBs consist of two domains, namely, an N-terminal ssDNA-binding/oligomerization domain and a flexible C-terminal protein-protein interaction domain without a defined tertiary structure [3, 29]. Tyrosine phosphorylation of SSB increases binding to ssDNA by almost 200-fold in vitro [30, 31]. The N-terminal domain is separated from the highly conserved acidic tail of the last 10 C-terminal amino acid residues of SSB by a long proline- or glycine-rich hinge [3, 32]. SSB interacts with other auxiliary proteins that are essential for cell survival [33]. The C-terminal acidic tail of SSB, such as “DDDIPF,” has been shown to bind to more than a dozen different proteins and the activity of some of these proteins is stimulated by their interactions with ssDNA-bound SSB [3]. The binding of SSB to ssDNA makes the glycine-rich region more easily accessible to other proteins such as proteases and DNA polymerase III [33, 34]. The C-terminus in SSB can also interact with the OB fold and regulate the ssDNA-binding activity of SSB itself [35, 36].

Studies on SSB from different organisms have grown rapidly during the past few years and knowledge on how SSBs interact with ssDNA has increased [22, 32, 37–46]. The most thoroughly studied SSB is that of* Escherichia coli* (EcSSB), which binds cooperatively to ssDNA [47]. The estimated binding site size of EcSSB is dependent on the salt concentration in fluorescence titrations with poly(dT) [47]. EcSSB mainly binds to 35- and 65-nucleotide- (nt) long ssDNA via the (SSB)_35_- and (SSB)_65_-binding modes, respectively. In the (SSB)_35_-binding mode, two subunits of the EcSSB tetramer interact with ssDNA, whereas in the (SSB)_65_-binding mode all four subunits participate in ssDNA binding. These different binding modes may be required during different stages of DNA metabolism for the in vivo function of SSB [48–50]. Although SSB binds to ssDNA via the highly conserved ssDNA-binding domain, the reason that the binding site sizes of SSBs from different organisms differ remains unclear. For example, differences are found among the binding site sizes of* Methanococcus jannaschii* SSB [51], the Gonococcal Genetic Island-encoded SSB from* Neisseria gonorrhoeae* [39], the thermostable* Thermotoga maritima* and* Thermotoga neapolitana* SSBs [32], and the psychrophilic bacterial SSBs [37]. In addition, the (SSB)_35_- and (SSB)_65_-binding modes are not found in some SSBs [32, 39, 42].

Previously, we have examined the electrophoretic mobility shift patterns of a His-tagged* Klebsiella pneumoniae* SSB (KpSSB) [40], a His-tagged* Salmonella enterica* serovar Typhimurium LT2 SSB (StSSB) [43], and a His-tagged* Pseudomonas aeruginosa* PAO1 SSB (PaSSB) [42] bound to different lengths of ssDNA. We also determined their corresponding binding site sizes, that is, 26, 22, and 29 nt per tetramer, respectively. The electrophoretic mobility shift assay (EMSA) is a well-established approach in studies of molecular biology [52], and the use of radioactive tracer in this assay allows visualization of the actual formation of the distinct protein-DNA complex(es)[53]. The expected result of EMSA is that when the length of the nucleotides is sufficient for the binding of two or more SSB molecules, the electrophoretic mobility of the higher SSB oligomer complex will be lower than that of the smaller SSB oligomer complex [52, 54]. Recent studies on SSB binding also reveal that determination of the ssDNA-binding site size by using EMSA is significantly consistent with that of the cocrystal structure of SSB with ssDNA [27].

**Figure 1:**
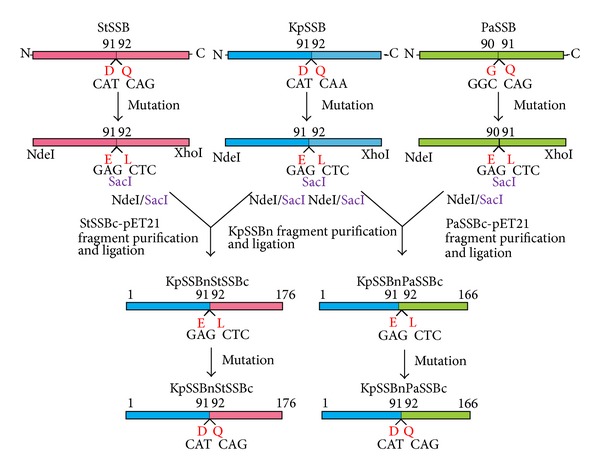
Construction of plasmids for expression of the chimeric KpSSBnStSSBc and KpSSBnPaSSBc proteins. To investigate the effect of the C-terminal domain of SSB on the size of the ssDNA-binding site, the C-terminal domain of KpSSB was replaced by that of StSSB and PaSSB. pET21b-KpSSB (Primers 7 and 8), pET21b-StSSB (Primers 9 and 10), and pET21b-PaSSB (Primers 11 and 12) vectors were mutated to create a desired SacI site and to obtain the vectors for expression of the chimeric proteins KpSSBnStSSBc and KpSSBnPaSSBc. The D91E/Q92L-engineered pET21b-KpSSB vector, the D91E/Q92L-engineered pET21b-StSSB vector, and the G90E/Q91L-engineered pET21b-PaSSB vector were cut at NdeI and SacI sites. Subsequently, the KpSSBn, StSSBc-pET21b, and PaSSBc-pET21b fragments were purified. KpSSBn was ligated with StSSBc-pET21b and PaSSBc-pET21b fragments to generate the engineered pET21b-KpSSBnStSSBc and pET21b-KpSSBnPaSSBc vectors. To avoid artificial residues, positions 91 and 92 of the two plasmids were mutated back (Primers 13 to 16) to obtain pET21b-KpSSBnStSSBc and pET21b-KpSSBnPaSSBc vectors. Thus, pET21b-KpSSBnStSSBc and pET21b-KpSSBnPaSSBc will express KpSSB1-91 fused StSSB92-176 and PaSSB91-165, respectively. Note that KpSSBnPaSSBc will have 166 amino acid residues.

KpSSB, StSSB, and PaSSB are similar proteins whose N-terminal ssDNA-binding domains are almost identical, except for different ssDNA-binding site sizes [40, 42, 43]. Thus, we should assess whether the glycine-rich hinge, which is not conserved among SSBs, is involved in the determination of the binding site size of SSB. In this study, we swapped the C-terminal domains of StSSB and PaSSB into that of KpSSB through protein chimeragenesis. Chimeras are proteins that contain segments from two or more different parent proteins and serve as valuable tools to understand enzyme mechanism and protein function [55]. The EMSA behavior (patterns) of the resultant chimeric proteins, namely, KpSSBnStSSBc and KpSSBnPaSSBc, was characterized and compared with untagged KpSSB, StSSB, and PaSSB (Figure 1). On the basis of the chimeragenesis results, the flexible C-terminal domain of SSB was found to be involved in determining the ssDNA-binding site sizes.

## 2. Materials and Methods

### 2.1. Materials

All restriction enzymes and DNA-modifying enzymes were purchased from New England Biolabs (Ipswich, MA, USA) unless explicitly stated otherwise. All chemicals were purchased from Sigma-Aldrich (St. Louis, MO, USA) unless explicitly stated otherwise. The* E. coli* strains TOP10F′ (Invitrogen, USA) and BL21(DE3)pLysS (Novagen, UK) were used for genetic construction and protein expression, respectively.

### 2.2. Construction of Plasmids for KpSSB, StSSB, and PaSSB Expression

The KpSSB [40], StSSB [43], and PaSSB [42] expression plasmids were constructed by the protocols described previously, with minor modification, to avoid having a His tag fused with the gene product. A fragment containing the coding sequence of KpSSB (*KPN04446*), StSSB (*STM4256*), and PaSSB (*PA4232*) (with the stop codon) was directly amplified by PCR by using the genomic DNA of* K. pneumoniae* subsp.* pneumoniae* MGH 78578,* S. enterica* serovar Typhimurium LT2, or* P. aeruginosa* PAO1 (Primers 1 to 6, resp.). During the process, NdeI and XhoI restriction sites were introduced at the 5′-end and the 3′-end of these genes, after which they were ligated into the pET21b vector (Novagen Inc., Madison, WI, USA) for protein expression in* E. coli* BL21. The expected gene product expressed by these plasmids does not contain any artificial residue, including a His tag. Primers used for construction of these plasmids are summarized in Table 1.

### 2.3. Construction of Plasmids for KpSSBnStSSBc and KpSSBnPaSSBc Expression through Protein Chimeragenesis

To investigate the effect of the C-terminal domain of SSB on the size of the ssDNA-binding site, the C-terminal domain of KpSSB was replaced by that of StSSB and PaSSB. pET21b-KpSSB (Primers 7 and 8), pET21b-StSSB (Primers 9 and 10), and pET21b-PaSSB (Primers 11 and 12) vectors were mutated to create a desired SacI site and to obtain the vectors for expression of the chimeric proteins KpSSBnStSSBc and KpSSBnPaSSBc. The D91E/Q92L-engineered pET21b-KpSSB vector, the D91E/Q92L-engineered pET21b-StSSB vector, and the G90E/Q91L-engineered pET21b-PaSSB vector were cut at NdeI and SacI sites. Subsequently, the KpSSBn, StSSBc-pET21b, and PaSSBc-pET21b fragments were purified. KpSSBn was ligated with StSSBc-pET21b and PaSSBc-pET21b fragments to generate the engineered pET21b-KpSSBnStSSBc and pET21b-KpSSBnPaSSBc vectors. To avoid artificial residues, positions 91 and 92 of the two plasmids were mutated back (Primers 13 to 16) to obtain pET21b-KpSSBnStSSBc and pET21b-KpSSBnPaSSBc vectors. Thus, pET21b-KpSSBnStSSBc and pET21b-KpSSBnPaSSBc will express KpSSB1-91 fused StSSB92-176 and PaSSB91-165, respectively. Note that KpSSBnPaSSBc will have 166 amino acid residues. Plasmids were verified by DNA sequencing. Underlined nucleotides indicate the designated site for mutation or the restriction site (Table 1).

### 2.4. Protein Expression and Purification

The recombinant SSBs were expressed using the protocol described previously [9, 40, 42, 43, 56–60]. Purification of these recombinant SSBs was carried out as described previously with the following modifications [61, 62]. Briefly,* E. coli* BL21(DE3) cells were individually transformed with the expression vector and grown to OD_600_ of 0.9 at 37°C in Luria-Bertani medium containing 250 *μ*g/mL ampicillin with rapid shaking. Overexpression of the expression plasmids was induced by incubating with 1 mM isopropyl thiogalactoside (IPTG) for 3 h at 37°C. The cells overexpressing the protein were chilled on ice, harvested by centrifugation, resuspended in Buffer A (20 mM Tris-HCl, 5 mM imidazole, and 0.2 M ammonium sulfate, pH 7.9), and disrupted by sonication with ice cooling. The protein solution (50 mL) was precipitated from the supernatant of the cell lysate by incubation with 0.27 g/mL of ammonium sulfate for 30 min and centrifugation at 20000 g for 10 min. The pellets were washed twice with 2.0 mL of Buffer B (20 mM Tris-HCl, 5 mM imidazole, and 1.2 M ammonium sulfate, pH 7.9). After dialysis against Buffer C (20 mM Tris-HCl, 5 mM imidazole, 1 mM EDTA, and 100 mM NaCl, pH 7.9), the protein solution applied to the Q column (GE Healthcare Bio-Sciences, Piscataway, NJ, USA) was eluted with a linear NaCl gradient from 0.1 to 0.6 M with Buffer C using the AKTA-FPLC system (GE Healthcare Bio-Sciences, Piscataway, NJ, USA). The peak fractions with the ssDNA-binding activity were collected and dialyzed against Buffer D (20 mM potassium phosphate, 1 mM EDTA, and 100 mM NaCl, pH 7.0). The protein solution was then applied to the Heparin HP column (GE Healthcare Bio-Sciences, Piscataway, NJ, USA) and eluted with a linear NaCl gradient from 0.1 to 1.0 M with Buffer D. The peak fractions from this chromatographic step with the ssDNA-binding activity were collected and concentrated, and the purity of these SSBs was checked by Coomassie-stained SDS-PAGE (Mini-PROTEAN Tetra System, Bio-Rad, CA, USA; Figure 3).

### 2.5. Protein Concentration

The protein concentration of the solutions was determined by the Bio-Rad Protein Assay using bovine serum albumin as a standard (Bio-Rad, CA, USA). The Bio-Rad Protein Assay is a dye-binding assay in which a differential color change of a dye occurs in response to various concentrations of protein.

### 2.6. Gel-Filtration Chromatography

Gel-filtration chromatography was carried out by the AKTA-FPLC system (GE Healthcare Bio-Sciences, Piscataway, NJ, USA). Briefly, purified protein (2 mg/mL) was applied to a Superdex 200 HR 10/30 column (GE Healthcare Bio-Sciences, Piscataway, NJ, USA) equilibrated with Buffer D. The column was operated at a flow rate of 0.5 mL/min, and 0.5 mL fractions were collected. The proteins were detected by measuring the absorbance at 280 nm. The column was calibrated with proteins of known molecular weight: thyroglobulin (670 kDa), *γ*-globulin (158 kDa), ovalbumin (44 kDa), and myoglobin (17 kDa). The *K*_av_ values for the standard proteins and the SSB variants were calculated from the equation: *K*_av_ = (*V*_*e*_ − *V*_*o*_)/(*V*_*c*_ − *V*_*o*_), where *V*_*o*_ is column void volume, *V*_*e*_ is elution volume, and *V*_*c*_ is geometric column volume.

### 2.7. Electrophoretic Mobility Shift Assay (EMSA)

EMSA [52] for these SSBs was carried out by the protocol described previously for DnaB [63], PriB [59, 64–66], DnaT [57, 67], and SSB proteins [40, 42, 43, 52]. Briefly, radiolabeling of various lengths of ssDNA oligonucleotides was carried out with [*γ* ^32^P]ATP (6000 Ci/mmol; PerkinElmer Life Sciences, Waltham, MA) and T4 polynucleotide kinase (Promega, Madison, WI, USA). The protein (0, 19, 37, 77, 155, 310, 630, 1250, 2500, and 5000 nM) was incubated for 30 min at 25°C with 1.7 nM DNA substrates (dT15–65) in a total volume of 10 *μ*L in 20 mM Tris-HCl pH 8.0 and 100 mM NaCl. Aliquots (5 *μ*L) were removed from each of the reaction solutions and added to 2 *μ*L of gel-loading solution (0.25% bromophenol blue and 40% sucrose). The resulting samples were resolved on a native 8% polyacrylamide gel at 4°C in TBE buffer (89 mM Tris borate and 1 mM EDTA) for 1 h at 100 V and were visualized by autoradiography. Complexed and free DNA bands were scanned and quantified.

### 2.8. DNA-Binding Ability

The ssDNA-binding ability ([Protein]_50_; *K*_*d*,app_) for the protein was estimated from the protein concentration that binds 50% of the input ssDNA [52]. Each [Protein]_50_ is calculated as the average of three measurements ± SD.

### 2.9. Bioinformatics

Sequence alignment of KpSSB, StSSB, and PaSSB was generated by CLUSTALW2 [68]. The structure of the C-terminal domain of these SSBs was modeled by (PS)^2^ (http://140.113.239.111/~ps2v2/docs.php/). The structures were visualized by using the program PyMol.

## 3. Results

### 3.1. Sequence Analysis

**Figure 2:**
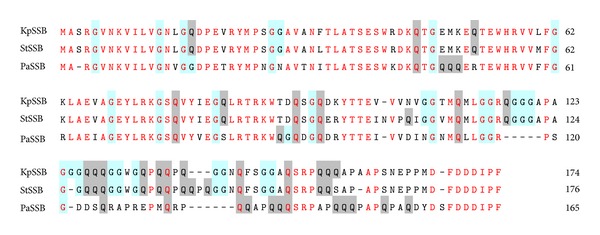
Multiple amino acid sequence alignment of SSB proteins. Sequence alignment of KpSSB, StSSB, and PaSSB was generated by CLUSTALW2. Identical amino acid residues are colored in red. Gly and Gln residues are shaded in cyan and gray. The N-terminal domains of these SSBs are significantly conserved.

**Figure 3:**
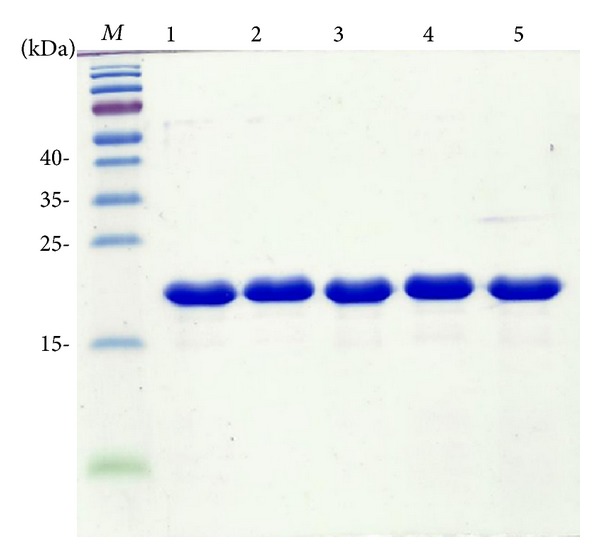
Protein purity. Coomassie Blue-stained SDS-PAGE (15%) of the purified KpSSB (lane 1), StSSB (lane 2), PaSSB (lane 3), KpSSBnStSSBc (lane 4), KpSSBnPaSSBc (lane 5), and molecular mass standards (*M*) are shown. The sizes of the standard proteins, from the top down, are as follows: 55, 40, 35, 25, 15, and 10 kDa. The purified SSBs migrated between the 25 and 15 kDa standards on the SDS-PAGE.

Based on the nucleotide sequence found, using a database search through the National Center for Biotechnology Information (NCBI), we predicted that KpSSB, StSSB, and PaSSB monomer proteins have lengths of 174, 176, and 165 amino acid residues, respectively. The size of the ssDNA-binding site of His-tagged KpSSB [40], StSSB [43], and PaSSB [42] was determined to be 26 ± 1, 22 ± 1, and 29 ± 1 nt, respectively. The longer the length of the polypeptide chain, the smaller the size for ssDNA binding. Analysis of the primary structures of KpSSB, StSSB, and PaSSB by RPS-BLAST revealed the presence of a putative OB-fold domain that is common to all known SSBs.  Figure 2 shows that the alignments of the amino acid sequences of KpSSB, StSSB, and PaSSB amino acid residues in their N-terminal domains are highly conserved (colored in red). In the* E. coli* SSB-ssDNA complex [11], four essential aromatic residues, namely, Trp40, Trp54, Phe60, and Trp88, participate in ssDNA binding via stacking interactions [11]. These residues are conserved in most SSB families, including KpSSB, StSSB, and PaSSB. The important motif in the C-terminal tail of* E. coli* SSB, DDDIPF residues, is also conserved in KpSSB, StSSB, and PaSSB. By contrast to those motifs, the residues found in the glycine-rich hinge of* E. coli* SSB are not conserved in KpSSB, StSSB, and PaSSB (Figure 2). Thus, the length and composition of the amino acid residues in the glycine-rich hinge may be responsible for the different ssDNA-binding site sizes of SSBs.

### 3.2. Expression and Purification of KpSSB, StSSB, and PaSSB

The N-terminal ssDNA-binding domain of SSB has been well-established to be highly conserved. However, SSBs possessing different ssDNA-binding site sizes have been reported. The reason that SSBs have similar ssDNA-binding domains but possess varying ssDNA-binding site sizes remains unclear. Although the ssDNA-binding site sizes of KpSSB, StSSB, and PaSSB have been reported, we reinvestigated the ssDNA-binding properties of KpSSB, StSSB, and PaSSB in the absence of a His tag to avoid the unknown effect of a His tag (hexahistidine) on the ssDNA binding of SSB.

### 3.3. KpSSB Bound to ssDNA

**Figure 4:**
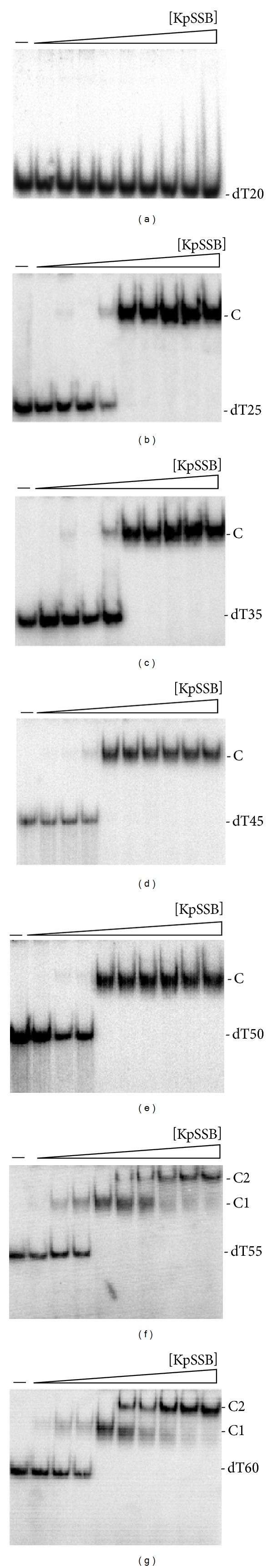
Binding of KpSSB to dT20–60. KpSSB (0, 19, 37, 77, 155, 310, 630, 1250, 2500, and 5000 nM) was incubated for 30 min at 25°C with 1.7 nM of (a) dT20, (b) dT25, (c) dT35, (d) dT45, (e) dT50, (f) dT55, or (g) dT60 in a total volume of 10 *μ*L in 20 mM Tris-HCl pH 8.0 and 100 mM NaCl. Aliquots (5 *μ*L) were removed from each reaction solution and added to 2 *μ*L of gel-loading solution (0.25% bromophenol blue and 40% sucrose). The resulting samples were resolved on a native 8% polyacrylamide gel at 4°C in TBE buffer (89 mM Tris borate and 1 mM EDTA) for 1 h at 100 V and visualized by autoradiography. Complexed and free DNA bands were scanned and quantified.

To investigate the length of nucleotides sufficient for the formation of the KpSSB-ssDNA complex and the ssDNA-binding ability of KpSSB, we studied the binding of KpSSB to dT20 (Figure 4(a)), dT25 (Figure 4(b)), dT35 (Figure 4(c)), dT45 (Figure 4(d)), dT50 (Figure 4(e)), dT55 (Figure 4(f)), and dT60 (Figure 4(g)) with different protein concentrations. As shown in Figure 4(a), no band shift was observed when KpSSB was incubated with dT20, indicating that KpSSB could not form a stable complex with this homopolymer. By contrast to dT20, longer dT homopolymers, which include dT25–50, produced a significant band shift (C, complex), that is, formation of a stable protein-DNA complex in solution. Furthermore, two different complexes for dT55 were formed by KpSSB (Figure 4(f)). At lower protein concentrations, KpSSB formed a single complex (C1) with dT55, similar to that observed with dT50 (Figure 4(e)). However, when the KpSSB concentration was increased, another slower migrating complex (C2) was observed. Two different complexes of KpSSB were also observed to bind to dT60 (Figure 4(g)). The appearance of the second complex resulted from the increased KpSSB concentration, suggesting that two KpSSB proteins may be present per oligonucleotide. Although dT55 is only 5 nt longer than dT50 is, the presence of an extra 5 nt in dT55 compared with that of dT50 provides enough interaction space for the binding of two KpSSB proteins. Therefore, one KpSSB occupies 25 (50/2 = 25) nt to 27.5 (55/2 = 27.5) nt of the ssDNA. The EMSA results suggest that the length of an ssDNA (or the binding site size) [52] required for KpSSB binding is 26 ± 2 nt.

### 3.4. StSSB Bound to ssDNA

**Figure 5:**
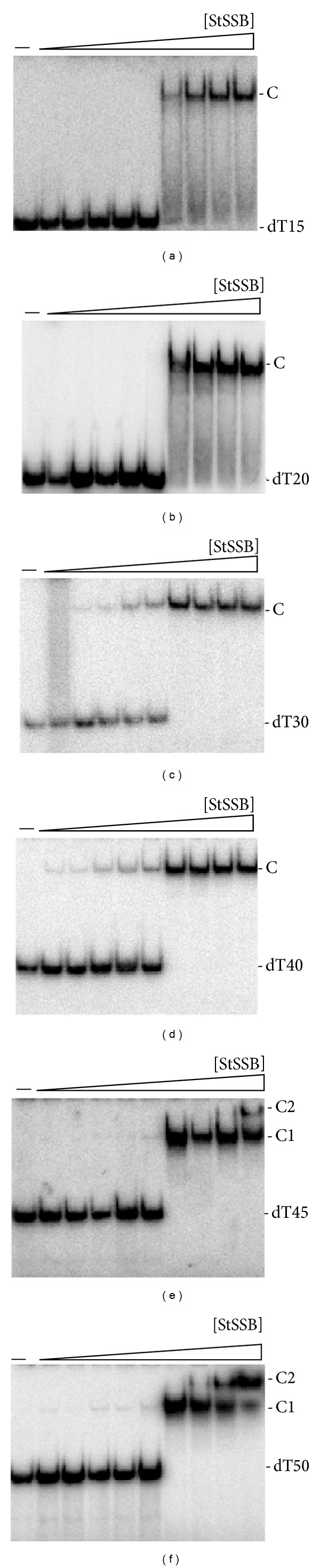
Binding of StSSB to dT15–50. StSSB (0, 19, 37, 77, 155, 310, 630, 1250, 2500, and 5000 nM) was incubated for 30 min at 25°C with 1.7 nM of (a) dT15, (b) dT20, (c) dT30, (d) dT40, (e) dT45, or (f) dT50 in a total volume of 10 *μ*L in 20 mM Tris-HCl pH 8.0 and 100 mM NaCl. Aliquots (5 *μ*L) were removed from each reaction solution and added to 2 *μ*L of gel-loading solution (0.25% bromophenol blue and 40% sucrose). The resulting samples were resolved on a native 8% polyacrylamide gel at 4°C in TBE buffer (89 mM Tris borate and 1 mM EDTA) for 1 h at 100 V and visualized by autoradiography. Complexed and free DNA bands were scanned and quantified.

The binding of StSSB to dT15 (Figure 5(a)), dT20 (Figure 5(b)), dT30 (Figure 5(c)), dT40 (Figure 5(d)), dT45 (Figure 5(e)), and dT50 (Figure 5(f)) was examined using EMSA. StSSB can bind and form a single complex with dT15 (Figure 5(a)) and dT20 (Figure 5(b)), but KpSSB cannot (Figure 4(a)). StSSB bound to dT15–40 and formed a single complex. For dT45 and dT50, two different complexes of StSSB appeared at high protein concentrations (Figures 5(e) and 5(f)). Therefore, one StSSB occupies 20 (40/2 = 20) nt to 22.5 (45/2 = 22.5) nt of the ssDNA. The EMSA results suggest that the length of an ssDNA (or the binding site size) [52] required for StSSB binding is 21 ± 2 nt.

### 3.5. PaSSB Bound to ssDNA

**Figure 6:**
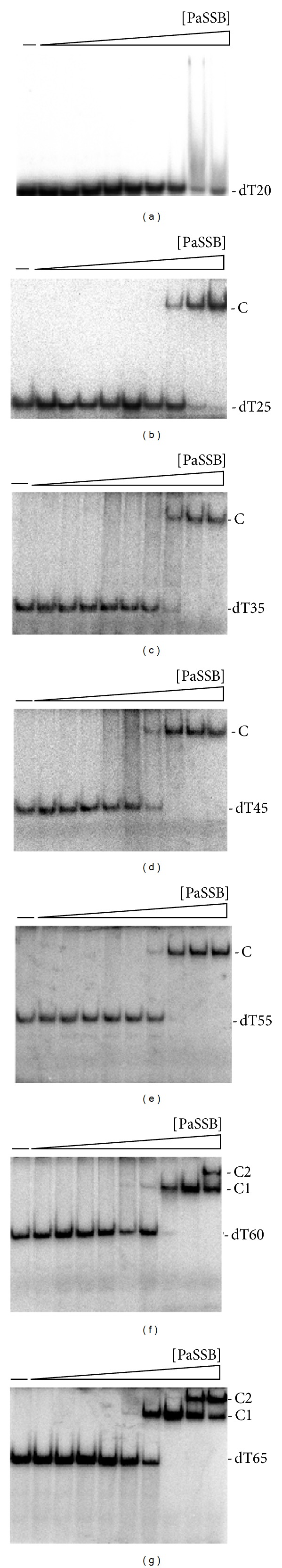
Binding of PaSSB to dT20–65. PaSSB (0, 19, 37, 77, 155, 310, 630, 1250, 2500, and 5000 nM) was incubated for 30 min at 25°C with 1.7 nM of (a) dT20, (b) dT25, (c) dT35, (d) dT45, (e) dT55, (f) dT60, or (g) dT65 in a total volume of 10 *μ*L in 20 mM Tris-HCl pH 8.0 and 100 mM NaCl. Aliquots (5 *μ*L) were removed from each reaction solution and added to 2 *μ*L of gel-loading solution (0.25% bromophenol blue and 40% sucrose). The resulting samples were resolved on a native 8% polyacrylamide gel at 4°C in TBE buffer (89 mM Tris borate and 1 mM EDTA) for 1 h at 100 V and visualized by autoradiography. Complexed and free DNA bands were scanned and quantified.

The binding of PaSSB to dT20 (Figure 6(a)), dT25 (Figure 6(b)), dT35 (Figure 6(c)), dT45 (Figure 6(d)), dT55 (Figure 6(e)), dT60 (Figure 6(f)), and dT65 (Figure 6(g)) was studied by EMSA. Unlike StSSB, no complex was observed when PaSSB was incubated with dT20. Some smears were observed, indicating that PaSSB interacts with dT20. However, the ssDNA may be too short to be fully wrapped by PaSSB. PaSSB could form a single complex with dT25–55 and form two distinct complexes with dT60 and dT65 (Figures 6(f) and 6(g)), respectively. Therefore, one PaSSB occupies 27.5 (55/2 = 27.5) nt to 30 (60/2 = 30) nt of the ssDNA. These results from EMSA suggest that the length of an ssDNA (or the binding site size) [52] required for PaSSB binding is 29 ± 2 nt. Although the SSBs, that is, KpSSB, StSSB, and PaSSB, have significantly similar ssDNA-binding domains, their binding site sizes are different and range from 19 (21 ± 2; StSSB) to 31 (29 ± 2; PaSSB) nt. The obtained EMSA results (Figures 4–6) also show that the binding site sizes of the untagged SSBs (KpSSB, StSSB, and PaSSB) were found to be almost identical to those of the His-tagged ones [40, 42, 43].

### 3.6. Design of the Chimeric KpSSB Proteins KpSSBnStSSBc and KpSSBnPaSSBc

The N-terminal ssDNA-binding domain of KpSSB, StSSB, and PaSSB is highly conserved (Figure 2), but their binding site sizes are different (Figures 4–6) and range from 19 nt to 31 nt. The C-terminal acidic tails, DDDIPF, are conserved (Figure 2), and these features led us to assess whether the flexible glycine-rich hinge in the C-terminal domain, which is not conserved among SSBs, is involved in the determination of the binding site size of SSB. Thus, the C-terminal domains of StSSB and PaSSB were swapped with KpSSB through protein chimeragenesis.

### 3.7. KpSSBnStSSBc Bound to ssDNA

**Figure 7:**
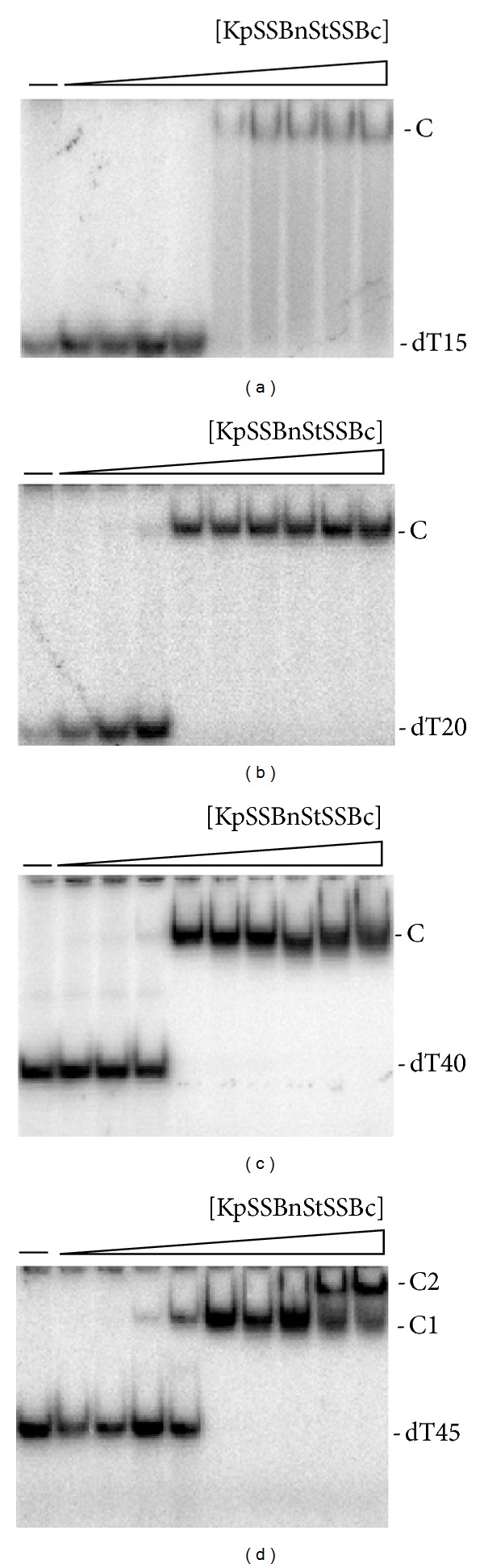
Binding of KpSSBnStSSBc to dT15–45. KpSSBnStSSBc (0, 19, 37, 77, 155, 310, 630, 1250, 2500, and 5000 nM) was incubated for 30 min at 25°C with 1.7 nM of (a) dT15, (b) dT20, (c) dT40, or (d) dT45 in a total volume of 10 *μ*L in 20 mM Tris-HCl pH 8.0 and 100 mM NaCl. Aliquots (5 *μ*L) were removed from each reaction solution and added to 2 *μ*L of gel-loading solution (0.25% bromophenol blue and 40% sucrose). The resulting samples were resolved on a native 8% polyacrylamide gel at 4°C in TBE buffer (89 mM Tris borate and 1 mM EDTA) for 1 h at 100 V and visualized by autoradiography. Complexed and free DNA bands were scanned and quantified.

**Figure 8:**
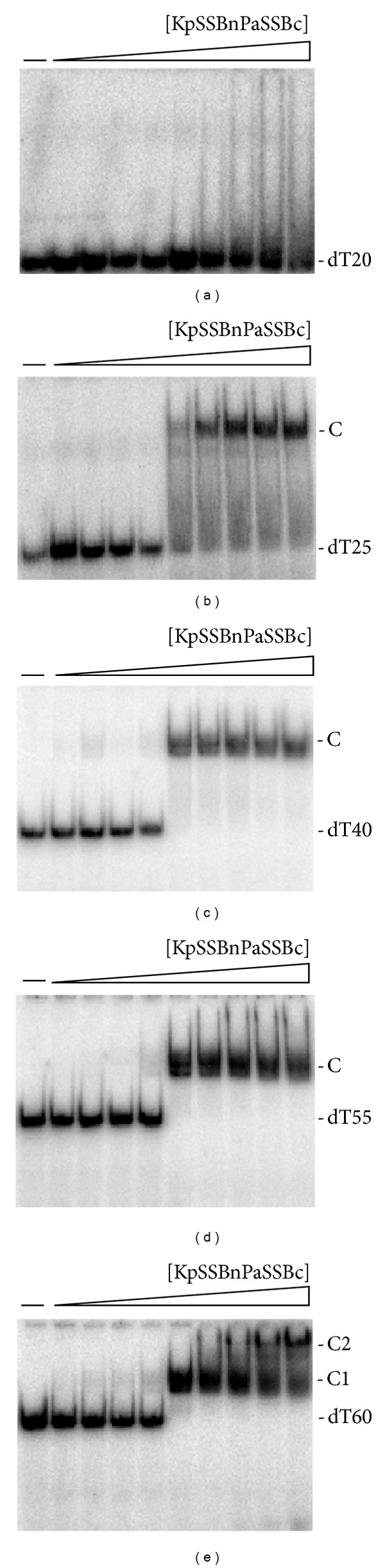
Binding of KpSSBnPaSSBc to dT20–60. KpSSBnPaSSBc (0, 19, 37, 77, 155, 310, 630, 1250, 2500, and 5000 nM) was incubated for 30 min at 25°C with 1.7 nM of (a) dT20, (b) dT25, (c) dT40, (d) dT55, or (e) dT60 in a total volume of 10 *μ*L in 20 mM Tris-HCl pH 8.0 and 100 mM NaCl. Aliquots (5 *μ*L) were removed from each reaction solution and added to 2 *μ*L of gel-loading solution (0.25% bromophenol blue and 40% sucrose). The resulting samples were resolved on a native 8% polyacrylamide gel at 4°C in TBE buffer (89 mM Tris borate and 1 mM EDTA) for 1 h at 100 V and visualized by autoradiography. Complexed and free DNA bands were scanned and quantified.

The binding of KpSSBnStSSBc to dT15 (Figure 7(a)), dT20 (Figure 7(b)), dT40 (Figure 7(c)), and dT45 (Figure 7(d)) was examined using EMSA. KpSSBnStSSBc exhibited significantly different ssDNA-binding properties from those of KpSSB. Unlike KpSSB (Figure 4), both KpSSBnStSSBc (Figure 8) and StSSB (Figure 5) can bind and form a single complex with dT15 and dT20. Similar to StSSB, KpSSBnStSSBc binds to dT15–40 and forms a single complex. For dT45, two different complexes of KpSSBnStSSBc appeared at high protein concentrations (Figure 8(d)); this EMSA feature was also similar to that of StSSB. One KpSSBnStSSBc occupies 20 (40/2 = 20) nt to 22.5 (45/2 = 22.5) nt of the ssDNA. These EMSA results suggest that the length of an ssDNA (or the binding site size) [52] required for KpSSBnStSSBc binding is 21 ± 2 nt, a value identical to that for StSSB (Figure 5). Swapping of the C-terminal domain of StSSB with KpSSB changes the size of the ssDNA-binding site from 26 nt to 21 nt.

### 3.8. KpSSBnPaSSBc Bound to ssDNA

**Figure 9:**
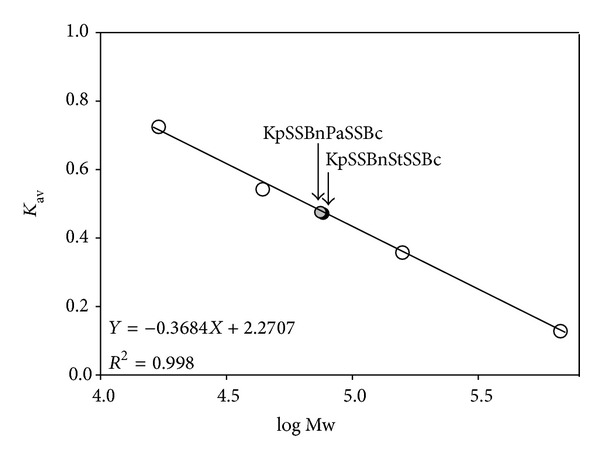
Gel-filtration chromatographic analyses of KpSSBnStSSBc and KpSSBnPaSSBc. Purified protein (2 mg/mL) was applied to a Superdex 200 HR 10/30 column (GE Healthcare Bio-Sciences, Piscataway, NJ, USA) equilibrated with Buffer D. The column was operated at a flow rate of 0.5 mL/min, and 0.5 mL fractions were collected. The proteins were detected by measuring the absorbance at 280 nm. The column was calibrated with proteins of known molecular weight: thyroglobulin (670 kDa), *γ*-globulin (158 kDa), ovalbumin (44 kDa), and myoglobin (17 kDa). The *K*_av_ values for the standard proteins and the SSB variants were calculated from the equation: *K*_av_ = (*V*_*e*_ − *V*_*o*_)/(*V*_*c*_ − *V*_*o*_), where *V*_*o*_ is column void volume, *V*_*e*_ is elution volume, and *V*_*c*_ is geometric column volume.

The binding features of KpSSBnPaSSBc with dT20 (Figure 8(a)), dT25 (Figure 8(b)), dT40 (Figure 8(c)), dT55 (Figure 8(d)), and dT60 (Figure 8(e)) were studied by EMSA. Similar to the cases of KpSSB and PaSSB, no complex was observed when KpSSBnPaSSBc was incubated with dT20. However, KpSSBnPaSSBc still exhibited dramatically different ssDNA-binding properties from those of KpSSB. KpSSB can form two distinct complexes with dT55 (Figure 4(f)), but both KpSSBnPaSSBc (Figure 9) and PaSSB (Figure 6) cannot. One KpSSBnPaSSBc occupies 27.5 (55/2 = 27.5) nt to 30 (60/2 = 30) nt of the ssDNA. The above EMSA results suggest that the length of an ssDNA (or the binding site size) [52] required for KpSSBnPaSSBc binding is 29 ± 2 nt, a value identical to that of PaSSB. Swapping of the C-terminal domain of PaSSB to KpSSB changes the size of the ssDNA-binding site from 26 nt to 29 nt. Although these SSBs, namely, KpSSB, StSSB, PaSSB, KpSSBnStSSBc, and KpSSBnPaSSBc, have nearly identical ssDNA-binding domains, their binding site sizes are different (Table 2). Thus, the size of the ssDNA-binding site required for second SSB binding is likely to be dependent on the C-terminal domain of SSB.

### 3.9. Binding Constants of the SSB-ssDNA Complexes Determined from EMSA

To compare the ssDNA-binding abilities of KpSSB, StSSB, PaSSB, KpSSBnStSSBc, and KpSSBnPaSSBc, the midpoint values for input ssDNA binding, calculated from the titration curves of EMSA and referred to as [Protein]_50_ (monomer), were quantified and are summarized in Table 2. Although the N-terminal ssDNA-binding domains of these SSB proteins are highly similar (Figure 2), their ssDNA-binding activities and binding site sizes are different (Table 2). [KpSSB]_50_ values ranged from 100 nM to 220 nM; [StSSB]_50_ values ranged from 420 nM to 650 nM; [PaSSB]_50_ values ranged from 550 nM to 1700 nM; [KpSSBnStSSBc]_50_ values ranged from 110 nM to 260 nM; and [KpSSBnPaSSBc]_50_ values ranged from 220 nM to 390 nM. The ssDNA-binding ability is as follows, in the order of decreasing affinity: KpSSB > KpSSBnStSSBc > KpSSBnPaSSBc > StSSB > PaSSB. Results from the above analyses indicate that the exchange of the C-terminal domain in SSB significantly changed the ssDNA-binding ability and the DNA-binding behavior (complex number). The reason as to why swapping of the C-terminal domain can affect the ssDNA-binding activity of SSB remains unclear. The C-terminal domain of SSB is suggested to be involved in ssDNA binding. However, this relation is not evident in the results of the cocrystal structure.

### 3.10. Oligomeric State of KpSSBnStSSBc and KpSSBnPaSSBc in Solution

Gel-filtration chromatography was used to confirm that the oligomeric state of KpSSBnStSSBc and KpSSBnPaSSBc remains as tetramers after chimeragenesis. The analysis of purified KpSSBnStSSBc and KpSSBnPaSSBc (2 mg/mL) using a Superdex 200 HR 10/30 column revealed a single peak with elution volumes of 78.6 and 78.9 mL, respectively. Assuming that KpSSBnStSSBc and KpSSBnPaSSBc both have shapes and partial specific volumes similar to the standard proteins, the native molecular masses of KpSSBnStSSBc and KpSSBnPaSSBc were estimated to be 76641 and 74827 Da, as calculated from a standard linear regression equation, *K*_av_ = −0.3684(logMw) + 2.2707 (Figure 9). The native molecular masses for KpSSBnStSSBc and KpSSBnPaSSBc are approximately four times the mass of the monomer (~19 kDa). Therefore, KpSSBnStSSBc and KpSSBnPaSSBc under the above chromatographic conditions are stable tetramers in solution. Although the exchange of the C-terminal domain in SSB significantly changed the ssDNA-binding ability and DNA-binding behavior (complex number), protein chimeragenesis did not cause any change in the oligomeric state of SSB.

### 3.11. Summary of Gly, Gln, and Pro Number in SSBs

To analyze the C-terminal amino acid composition of SSBs, we further counted the number of Gly, Gln, and Pro residues in different SSB segments. SSB is abundant in Gly, Gln, and Pro (GQP) (Table 3). The GQP contents of KpSSB1–91, StSSB1–91, and PaSSB1–90 are similar. However, the Gly number of PaSSB116–165 is significantly lower than that of KpSSB116–174 and StSSB117–176; PaSSB116–165 contains only 1 Gly, but KpSSB116–174 and StSSB117–176 contain 11 and 12 Gly, respectively. In addition, we found different distribution patterns among KpSSB, StSSB, and PaSSB. Although they contain similar number of Gln (Q), the QQQ pattern is frequently found in PaSSB (Table 3).

### 3.12. Structural Modeling of SSBs

**Figure 10:**
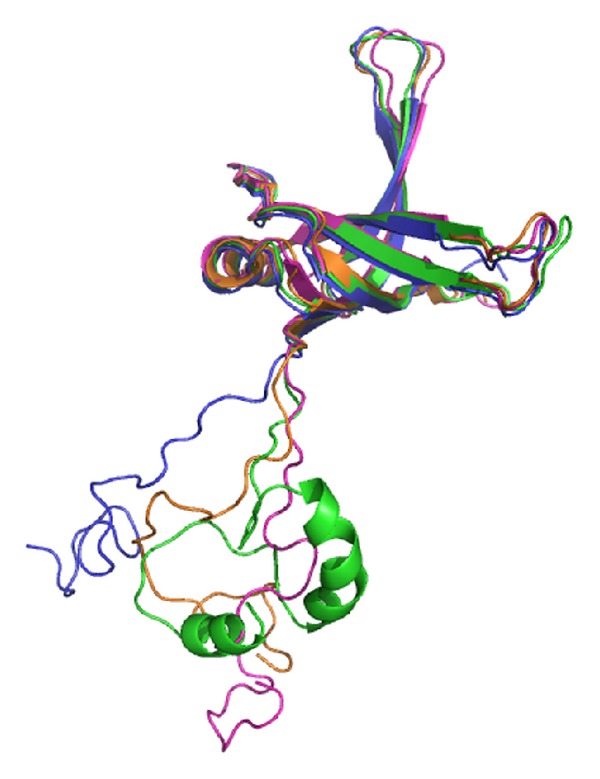
Structure modeling of SSB. The structures of KpSSB1–115, StSSB1–115, and PaSSB1–115 (the N-terminal domain of SSB) were modeled by SWISS-MODEL. The structures of KpSSB116–142, StSSB116–142, and PaSSB121–160 (the C-terminal domain of SSB) were modeled by (PS)^2^. Other regions of SSBs could not be modeled by these two programs. The structures of the N-terminal domain and the C-terminal domain of these SSBs were manually linked (KpSSB1–142, blue; StSSB1–142, pink; PaSSB1–160, green) and superimposed with the crystal structure of EcSSB1–142 (orange) (PDB entry: 1QVC) for comparison. For clarity, only one subunit of the tetramer was shown for each SSB.

Given its disordered C-terminal domain, the crystal structure of the full-length SSB is lacking, even when SSB can be crystallized with DNA [69]. We attempted to model the structure by homology modeling using the bioinformatics program (PS)^2^ to obtain an in-depth understanding of the structure-function relationship of the C-terminal domains of these SSBs [70, 71]. (PS)^2^ (http://140.113.239.111/~ps2v2/docs.php/) is an automatic homology modeling server that combines both sequence and secondary structure information to detect the homologous proteins with remote similarity and the target-template alignment. After pasting the amino acid sequence to the website of (PS)^2^, only one hit (Protein Data Bank entry: 1QVC; EcSSB) for the C-terminal domains of KpSSB and StSSB was suggested. For the C-terminal domain of PaSSB, only one hit, that is, CstF-77 (Protein Data Bank entry: 2OOE; cleavage stimulation factor, CstF), but not EcSSB, was suggested as the template for modeling.  Figure 10 shows that modeled structures of these SSB C-terminal domains are highly disordered but that of PaSSB is more ordered than that of other domains.

## 4. Discussion

In this study, we examined the sizes of the binding site of the untagged SSB and the chimeric SSB from the ubiquitous opportunistic pathogens* K. pneumoniae*,* S. enterica* serovar Typhimurium LT2, and* P. aeruginosa* PAO1. Many clinical strains of the abovementioned bacteria are highly resistant to antibiotics [72–75]. The development of clinically useful small-molecule antibiotics has been a seminal event in the field of infectious diseases [48]. Nucleic acid metabolism is one of the most basic biological functions and should be a prime target in antibiotic development [76–78]. Many bacterial SSBs form conserved protein interaction “hubs” that are essential to recruit many proteins involved in DNA replication, recombination, and repair SSB/DNA nucleoprotein substrates [79]. Thus, SSBs may be promising targets in antibiotic development [80]. As a first step toward achieving this goal, we investigated why SSBs possess highly conserved N-terminal ssDNA-binding domain but exhibit varying binding site sizes. One significant clue is that their flexible hinges and the length at the C-terminus are different as revealed by sequence alignment (Figure 2).

The interactions of various SSBs with ssDNA have been analyzed using a variety of techniques such as tryptophan-fluorescence quenching [47], filter binding [81], EMSA [52, 82], analytical ultracentrifugation [83], electron microscopy [84], nuclease digestion [44], single-molecule fluorescence microscopy [48], and crystallographic analyses [11]. In this study, we have examined the electrophoretic mobility shift patterns of KpSSB, StSSB, PaSSB, KpSSBnStSSBc, and KpSSBnPaSSBc bound to different lengths of ssDNA and determined the corresponding binding site sizes to be 26, 21, 29, 22, and 29 nt per tetramer, respectively (Figures 4–8). PaSSB and KpSSBnPaSSBc have the largest sizes for ssDNA binding among the SSBs studied. We also identified His-tagged and untagged SSBs that have similar ssDNA-binding site sizes [40, 42, 43]. EMSA is a well-established approach in studies of molecular biology [52], and the use of radioactive tracer in this assay allows detection of the actual formation of the distinct protein-DNA complex(es) [53]. For example, DNase protection assay and footprinting assay using radioactive tracer can determine the specific DNA sequence complexed by a protein. In EMSA, when the length of the nucleotides is sufficient for the binding of two or more SSB molecules, the electrophoretic mobility of the higher SSB oligomer complex will be lower than that of the smaller SSB oligomer complex [52, 54]. In addition, results of the ssDNA-binding site size from EMSA and cocrystal structure of SSB were consistent [27]. Thus, throughout this paper, we determined the ssDNA-binding site sizes of SSB from the EMSA behavior.

Many SSBs bind to ssDNA with some degree of positive cooperativity. Cooperativity can result from direct protein-protein interactions between the nearest neighbors, such as the LAST motif in the T4 gene-32 protein [85] and the arginine-mediated interaction motif in* Thermus* SSB [86, 87]. Cooperativity can also result from the protein-induced distortion of adjacent DNA, as demonstrated in* Sulfolobus* SSB, PriB, and FOXK1a proteins [23, 60, 88]. In the cases of KpSSB, StSSB, and PaSSB (Figures 4–6), binding appeared to be nearly noncooperative for several DNAs because all DNA mainly shifts into the first complex (C1) before the appearance of the second complex (C2) when subjected to increasing protein concentrations. The length dependence of the [SSB]_50_ values suggests that the amount of spacing is optimum for steric considerations (Table 2).

**Figure 11:**
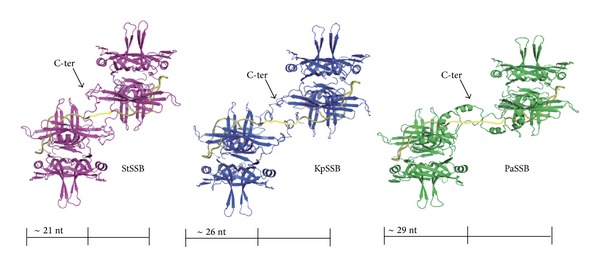
Possible models for explaining why SSBs are with different binding site sizes. Two modeled structures of KpSSB1–142 (blue), StSSB1–142 (pink), and PaSSB1–160 (green) complexed with ssDNA (gold) are shown. For clarity, only one C-terminal domain was shown for each SSB tetramer. By using the electrophoretic mobility shift assay and the protein chimeragenesis, we characterized that the binding site sizes of KpSSB, StSSB, PaSSB, KpSSBnStSSBc, and KpSSBnPaSSBc were 26, 21, 29, 21, and 29 nt per tetramer, respectively. KpSSB, StSSB, and PaSSB are similar proteins whose N-terminal ssDNA-binding domains are almost identical. Thus, the C-terminal domain of SSB may indirectly contribute to ssDNA binding and wrapping and affects the binding site size by the steric hindrance.

Because bacteria have varying genomic DNA sizes, their SSBs may need to evolve to have different binding site sizes for DNA metabolism. Results from protein chimeragenesis showed the C-terminal domain dependence of the binding site sizes of SSB (Figure 11). The experimental data showed that the binding site size of KpSSBnStSSBc was similar to that of StSSB and the size of the binding site of KpSSBnPaSSBc was similar to that of PaSSB. The reason for which the binding site size of SSB changed, followed by swapping of the C-terminal domain, remains unclear. Flexibility, number of glycine residues, and/or different QQQ patterns of the C-terminal domain of SSB (Figure 2 and Table 3) may be important factors for determining the ssDNA-binding site size. In fact, the C-terminal domain of PaSSB, that is, PaSSB116–165, has only 1 Gly residue, which is significantly less than that of KpSSB (11 Gly) and StSSB (12 Gly). Gly (and Pro) is an important component of the flexible region; a protein that contains low Gly content is predicted to have low flexibility. Unlike typical SSB [35, 69], PaSSB116–165 has a partial structure (Figure 10). Although KpSSB, StSSB, and PaSSB contain similar number of Gln (Q), the QQQ pattern is frequently found in PaSSB (Figure 2 and Table 3). PolyQ and repeated sequences GAGAG are commonly found in the structures of amyloids, silk fibers, and neurodegradation proteins [89–92]. Considering that the simple coil polyQ, the heptapeptide GNNQQNY, and the hexapeptide NNQQNY can cause protein aggregation and nucleation [93–95], the distribution of Gln in the C-terminal domain of a tetrameric SSB may also be an important determinant of the ssDNA-binding site size of SSB by some steric hindrances (Figure 11). However, the above speculation must be confirmed by further biochemical experiments.

## 5. Conclusion

In this study, we characterized the ssDNA-binding properties of untagged SSBs from* K. pneumoniae*,* S. enterica* serovar Typhimurium LT2, and* P. aeruginosa* PAO1 and proposed a role of the C-terminal flexible domain for ssDNA binding from the protein chimeragenesis and EMSA results. The amino acid sequence of the N-terminal ssDNA-binding/oligomerization domain in these pathogenic SSBs is highly conserved, but their apparent binding site sizes are different. This finding indicates that the C-terminal protein-protein interaction domain may also indirectly contribute to ssDNA binding and wrapping.

## Figures and Tables

**Table 1:** Primers used for construction of plasmids.

Oligonucleotide	Primer
1 KpSSB-NdeI-N	GGGCATATGGCCAGCAGAGGCGTAAAC
2 KpSSB-XhoI-C	GGGCTCGAGTTAGAACGGGATGTCGTC
3 StSSB-NdeI-N	CTGAACATATGGCCAGCAGAGGCGTAA
4 StSSB-XhoI-C	TGGAACTCGAGTTAGAACGGAATGTCG
5 PaSSB-NdeI-N	TTGCTCATATGGCCCGTGGGGTTAACA
6 PaSSB-XhoI-C	TTGCACTCGAGTTAGAACGGAATGTCG
7 KpSSB(D91E/Q92L-SacI)-N	AAGTGGACCGAGCTCTCCGGTCAGGACA
8 KpSSB(D91E/Q92L-SacI)-C	GTCCTGACCGGAGAGCTCGGTCCACTT
9 StSSB(D91E/Q92L-SacI)-N	AAGTGGACCGAGCTCAGTGGCCAGGAA
10 StSSB(D91E/Q92L-SacI)-C	TTCCTGGCCACTGAGCTCGGTCCACTT
11 PaSSB(G90E/Q91L-SacI)-N	AAGTGGCAGGAGCTCGACGGTCAGGAT
12 PaSSB(G90E/Q91L-SacI)-C	ATCCTGACCGTCGAGCTCCTGCCACTT
13 KpSSBnStSSBc(E91D/L92Q)-N	AAGTGGACCGATCAGAGTGGCCAGGAA
14 KpSSBnStSSBc(E91D/L92Q)-C	TTCCTGGCCACTCTGATCGGTCCACTT
15 KpSSBnPaSSBc(E91D/L92Q)-N	AAGTGGACCGATCAGGACGGTCAGGAT
16 KpSSBnPaSSBc(E91D/L92Q)-C	ATCCTGACCGTCCTGATCGGTCCACTT

A fragment containing the coding sequence of KpSSB, StSSB, and PaSSB (with the stop codon) was cloned into the pET21b vector (using Primers 1–6). During the process, NdeI and XhoI restriction sites were introduced at the 5′-end and the 3′-end of these genes, after which they were ligated into the pET21b vector. To obtain the vectors for expression of the chimeric proteins KpSSBnStSSBc and KpSSBnPaSSBc, pET21b-KpSSB (Primers 7 and 8), pET21b-StSSB (Primers 9 and 10), and pET21b-PaSSB (Primers 11 and 12) vectors were mutated to create a desired SacI site. The D91E/Q92L-engineered pET21b-KpSSB vector, the D91E/Q92L-engineered pET21b-StSSB vector, and the G90E/Q91L-engineered pET21b-PaSSB vector were cut at NdeI and SacI sites. Subsequently, the KpSSBn, StSSBc-pET21b, and PaSSBc-pET21b fragments were purified. KpSSBn was ligated with StSSBc-pET21b and PaSSBc-pET21b fragments to generate the engineered pET21b-KpSSBnStSSBc and pET21b-KpSSBnPaSSBc vectors. To avoid artificial residues, positions 91 and 92 of the two plasmids were mutated back (Primers 13 to 16) to obtain pET21b-KpSSBnStSSBc and pET21b-KpSSBnPaSSBc vectors. Thus, pET21b-KpSSBnStSSBc and pET21b-KpSSBnPaSSBc will express KpSSB1-91 fused StSSB92-176 and PaSSB91-165, respectively. These plasmids were verified by DNA sequencing. Underlined nucleotides indicate the designated site for mutation or the restriction site.

**Table 2:** ssDNA binding properties of KpSSB, StSSB, PaSSB, KpSSBnStSSBc, and KpSSBnPaSSBc as analyzed by EMSA.

Protein	DNA	[Protein]_50_ (nM)	Complex number
KpSSB	dT20	ND	0
	dT25	200 ± 20	1
	dT35	220 ± 30	1
	dT45	100 ± 10	1
	dT50	110 ± 20	1
	dT55	100 ± 20	2
	dT60	100 ± 10	2

StSSB	dT15	650 ± 120	1
	dT20	450 ± 80	1
	dT30	420 ± 60	1
	dT40	420 ± 80	1
	dT45	440 ± 60	2
	dT50	440 ± 50	2

PaSSB	dT20	ND	0
	dT25	1700 ± 250	1
	dT35	950 ± 180	1
	dT45	780 ± 160	1
	dT55	820 ± 90	1
	dT60	810 ± 110	2
	dT65	550 ± 70	2

KpSSBnStSSBc	dT15	260 ± 60	1
	dT20	110 ± 20	1
	dT40	120 ± 20	1
	dT45	160 ± 20	2

KpSSBnPaSSBc	dT20	ND	0
	dT25	390 ± 60	1
	dT40	220 ± 30	1
	dT55	230 ± 30	1
	dT60	230 ± 30	2

[Protein]_50_ was calculated from the titration curves of EMSA by determining the concentration of the protein (*μ*M) needed to achieve the midpoint value for input ssDNA binding. For some oligonucleotides, input ssDNA binding was the sum of the intensities from the two separate ssDNA-protein complexes. Errors are standard deviations determined by three independent titration experiments.

**Table 3:** Summary of Gly, Gln, and Pro number in SSB.

SSB segment	G	Q	P
KpSSB1–91	10	5	2
StSSB1–91	10	5	2
PaSSB1–90	11	6	2

KpSSB92–174	18	16	9
StSSB92–176	17	18	11
PaSSB91–165	5	15	11

KpSSB116–174	11	12	9
StSSB117–176	12	13	10
PaSSB116–165	1	12	11
